# Performance, carcass quality and meat quality of the endangered German Angler Saddleback pig

**DOI:** 10.1371/journal.pone.0300361

**Published:** 2024-04-30

**Authors:** Anna Olschewsky, Margret Krieger, Susanne Hoischen-Taubner, Daniel Mörlein, Stefanie Klingel, Georg Thaller, Dirk Hinrichs

**Affiliations:** 1 Animal Breeding Section, Faculty of Organic Agricultural Sciences, University of Kassel, Witzenhausen, Germany; 2 Department of Animal Nutrition and Animal Health, Faculty of Organic Agricultural Sciences, University of Kassel, Witzenhausen, Germany; 3 Department of Animal Sciences, University of Göttingen, Göttingen, Germany; 4 Center for Rare and Endangered Domestic Animals, Warder, Germany; 5 Institute of Animal Breeding and Husbandry, University of Kiel, Kiel, Germany; University of Agriculture Faisalabad, PAKISTAN

## Abstract

The Angler Saddleback pig is an endangered local breed originating from Germany. The breed is said to have low demands in terms of husbandry and feeding, and an excellent meat quality. To date, there is a lack of more recent scientific investigations of the breed. Therefore, 58 Angler Saddleback pigs were fattened in two consecutive trials whereby performance, carcass quality and meat quality were assessed. At an average age of 324 days, the pigs reached an average final live weight of 143 kg, an intramuscular fat (IMF) content of 2.6%, a lean meat percentage of 47% and a backfat thickness of 38 mm. A significant influence of the independent variables “breeder” and “age at the end of fattening” on the majority of target variables was found. Furthermore, IMF as well as pH value 45 minutes *post mortem* was significantly influenced by sex. These results give a current overview of the phenotypic characteristics of this endangered breed. It is shown that the slower growing Angler Saddleback breed may need alternative marketing concepts for its meat and meat products. Additionally, further research is necessary to clarify the reasons for the high phenotypic variation within this breed.

## Introduction

In Germany, pork is by far the most consumed meat [1]. Due to their high lean meat percentage (LMP), the use of commercial hybrid lines dominates the production of pig meat. Aside carcass weight, LMP is the decisive trait as per the currently used payment schemes of slaughterhouses in Germany [2]. Consequently, carcasses (and the meat) of slower growing local pig breeds like the Angler Saddleback pig are economically disadvantageous because of a higher fat amount [3]. However, higher fat contents, especially in the form of intramuscular fat (IMF), can positively influence taste and tenderness as important sensory qualities of pork [4, 5]. This might be one reason why the meat of Angler saddleback pigs is (said to be) valued by connoisseurs [3].

The Angler Saddleback pig is an endangered local breed, originating from the Northern part of Germany. Although this breed has a very low population size of 94 female and 26 male herdbook-registered pigs [6], lively activities of the Angler Saddleback breeders are documented. The breeders are organized in an association and an Angler Saddleback herdbook has been established since the year 1929 [3]. In contrast to the usual feeding practices on commercial pig farms, which are mainly based on concentrated feed, the Angler Saddleback breed is said to make good use of regional feeds including high amounts of roughage [7]. The ability to utilise alternative local feed resources could be an advantage in the context of rising feed prices. The Angler Saddleback breed is mainly kept in traditional smallholder and free-range systems, a more natural environment which could contribute to better animal welfare, and is generally considered robust and less demanding [8].

Altogether, the traditional husbandry system of Angler Saddleback pigs and the production of high-quality meat products are valuable for the region of origin. Furthermore, the breed’s less demanding nature in terms of feed and husbandry might be important in the future when livestock management may face challenges that are unknown today [9]. Therefore, it may be worthwhile to conserve this animal genetic resource. However, there is a lack of current scientific evidence going beyond genetic analyses and shedding light on the phenotypic characteristics of the breed. Evidence of its beneficial traits could contribute to the promotion and conservation of the breed.

Against this background, the present study focused on general performance, carcass and meat quality of Angler Saddleback pigs, wherein the following research questions where considered (i) What growth performance and carcass quality as well as meat quality do Angler Saddleback pigs achieve under (semi-)controlled conditions? (ii) Do batch, sex, breeder (origin of the piglets) and age influence target variables describing these performance and quality aspects?

To answer these questions, 60 Angler Saddleback pigs were reared and fattened, in two consecutive trials, on an experimental farm and parameters associated with growth performance, carcass quality and meat quality were assessed.

## Animals, materials and methods

### Ethic statement

The study was carried out in accordance with the German animal protection act [10]. The protocol for the investigations of performance, carcass and meat quality was reported to the Animal Welfare Officer of the University of Kiel without objection.

### Pigs and husbandry

In total, 60 purebred Angler Saddleback pigs, which descended from herdbook-registered parents, were reared and fattened in two consecutive trials. Due to the limited availability of piglets, 21 female and 19 castrated males were included in the first batch (October 2018 to May 2019), whereas the second batch (February 2020 to October 2020) consisted of 11 females and 9 castrated males. The animals came from five different breeders from Northern Germany. In the first batch the pigs from breeder 1 (n = 15), 2 (n = 15) and 3 (n = 10) were investigated and in the second batch from breeder 4 (n = 3) and 5 (n = 15). At the beginning of the fattening period, the pigs were between 66 and 147 days old. This was also a consequence of the insufficient availability of animals due to the overall low population size. In order to achieve similar live weights and to compensate for the different age of the animals, two slaughter dates were carried out per batch. Consequently, the pigs were slaughtered at an age between 282 and 377 days. Two death losses occurred in batch 2 (one male and one female), resulting in a total of 58 pigs in the final data set.

The husbandry was conducted in concordance with the Animal welfare—farm animal husbandry ordinance [11]. The pigs were kept in single compartments with solid flooring and straw bedding, and without control of temperature and ventilation (semi-controlled conditions). They were fed with a slightly energy and protein reduced commercial diet, divided into two phases and based mainly on wheat and rapeseed meal (Table 1). Daily concentrate amounts were restricted and ranged between 1.5 and 3.0 kg fresh matter (FM) per pig per day. The change from grower to finisher diet took place for each animal individually at a weight of 60 kg. By this, an excessive fat content in the meat was to be prevented and the previously described lower nutritional demands of the breed were met. In addition to the concentrated feed, grass-clover silage (2^nd^ and 3^rd^ cut) was offered to the animals with no further analysis of the nutrient composition. Silage amounts ranged from 1.5 to 3.0 kg FM per pig per day. Feed amounts were measured only for batch 2 and on a volume basis, i.e. buckets for concentrate and wheelbarrows for silage, and the average weight of each measure was determined at the start of the batch.

**Table 1:** Nutrient and energy content of the grower and finisher diets of batches 1 and 2, according to feed declaration, compared with standard rations for fattening pigs (based on 88% dry matter).

	Grower			Finisher		
Items	Batch 1	Batch 2	Standard	Batch 1	Batch 2	Standard
Crude protein (%)	16.0	16.0	16.0–18.0^**1**^	13.5	13.5	13.0–15.0^**1**^
Lysine (%)	0.90	0.90	0.95–1.02^**1**^	0.60	0.60	0.70–0.80^**1**^
Methionine (%)	0.28	0.28	0.25–0.28^**1**^	0.18	0.19	0.2^**1**^
Crude fiber (%)	5.30	5.30	>3.0^**1**^	7.00	7.00	>3.0^**1**^
Crude fat (%)	3.70	3.60		3.70	3.70	
Crude ash (%)	5.40	5.50		5.00	5.00	
Ca (%)	0.90	0.90	0.60–0.70^2^	0.65	0.65	0.50–0.55^2^
P (%)	0.55	0.55	0.45–0.50^2^	0.45	0.45	0.40^2^
Na (%)	0.23	0.23	0.10–0.15^2^	0.20	0.20	0.10^2^
ME (MJ)	12.6	12.6	13.0–13.4^2^	11.8	11.8	12.5–13.0^2^

^1^ Example mixatures for organic pig fattening at 750 g daily gain [12]

^2^ General reference values per kg fattening feed at 750g daily gain [12]

### Performance, carcass and meat quality measures

As two slaughterings were carried out per batch, only a small group of animals had to be transported to the slaughterhouse, respectively. Transportation distance was 25 km and the pigs were handled carefully by staff familiar with the pigs. The animals were slaughtered in accordance with the requirements of the EU regulation on the protection of animals at the time of killing [13] in a small countryside slaughterhouse. The pigs were individually stunned with an electrical tong followed by sticking and bleeding. Scalding and dehairing was done by machine with some manual finishing. The carcasses were directly transferred to the cold room and were chilled at 3°C until the next day. All *post mortem* measurements were conducted in the slaughterhouse by trained staff following the German “Richtlinie für die Stationsprüfung auf Mastleistung, Schlachtkörperwert und Fleischbeschaffenheit beim Schwein” [Guideline for station testing for fattening performance, carcass value and meat quality in pigs] [14]. For calculating performance traits, live weight at the start of the rearing and again before slaughter was used. The average daily weight gain per pig was computed as the quotient of summarized weight gain per animal and the total housing period (days). Carcass weight was determined directly after gutting. Dressing percentage was calculated by dividing the carcass weight by the live weight of the animal immediately before slaughter and expressing the result as a percentage. The cutting was conducted 24 hours after slaughter and the weight of the valuable parts were taken. Backfat thickness was calculated based on the measurement at three points (thickest part of the withers, thinnest part of the center of the back, thinnest overlay of the *M*. *glutaeus medius*). To assess meat quality, the pH-value was measured 45 minutes (pH_45_) and 24 hours (pH_24_) *post mortem* in the loin muscle (M. *Longissimus thoracis et lumborum*) between the 2nd and 3rd last rib and ham (*M*. *semimembranosus*), 6 cm above the aitch bone using the pH-Star device (Matthäus, Pöttmes, Germany). Electric conductivity was assessed 45 minutes and 24 hours *post mortem* in the loin muscle and ham using the LF-Star-device (Matthäus, Pöttmes, Germany). With the use of Opto-Star-device (Matthäus, Pöttmes, Germany), the brightness of the meat was measured on the cut and cleaned surface of the *M*. *longissimus*. Drip loss was assessed using the EZ-method and computed as the difference between the weight before and after a storage time of 24 hours [15]. Following Otto et al. (2004) [16], two meat samples were removed 24 hours *post mortem* from the 14^th^ rib of the loin muscle using a circular knife. The samples were weighed (Ø 12 g), stored in drip loss containers and transported, under cooled conditions, to the laboratory at the University of Kiel where they were re-weighed.

Based on the “two point” method, LMP estimates were calculated using the following formula: LMP = 58,10–0,56 x backfat measurement (including rind) in mm + 0,13 x meat measurement (thickness of loin muscle) in mm. Therefore, backfat thickness was measured at the thinnest point of the backfat above the *M*. *glutaeus medius*, and muscle depth was measured as the shortest horizontal junction of the anterior (cranial) end of *M*. *glutaeus medius* to the superior (dorsal) edge of the spinal canal [17].

Additionally, samples for determination of IMF and dry matter were taken from *M*. *longissimus* between the 13^th^ and 14^th^ rib. Under cooled conditions, they were transported to the laboratory of the University of Kassel where the analyses were performed using Near Infrared Spectroscopy (FOSS NIRSystems, Hamburg, Germany). Analyses were based on an in-house calibration (R^2^ = 0.89, RMSEP = 0.26, range of the validation = 1.93–4.47), with a reference method including the crude fat content (according to VDLUFA 5.1.1) and the dry matter (seasand method).

### Statistical analysis

Statistical analyses were conducted using R [18]. Because of distinct differences between the two batches (e.g. age at the start of the fattening and number of animals), descriptive analyses were done for each batch separately. Using the R package “FactoMineR” (v2.6, [19]), principal component analyses (PCA) of target variables and individuals were conducted and visualized with the package “factoextra” (v1.0.7, [20]). By default, the process of standardizing the variables is included in the “FactoMineR” package. Additionally, the possible influence of six independent variables (batch, breeder, sex, weight at the start of fattening and age at the start and end of fattening) on selected target variables of the domains growth performance, carcass quality and meat quality was assessed. Therefore, linear models were built using the lm() function. As a first step, the relationships between the regressors were tested for every model to avoid collinearity using mctest (v1.3.1, [21]). In all cases this resulted in a complete collinearity between the regressors ‘batch’ and ‘breeder 4’ and ‘breeder 5’. This result is plausible because breeders were split between batches and therefore the content of the two variables overlaps. Additionally, collinearity was detected for the independent variables ‘age at the start of fattening’ and ‘weight at the start of fattening’ against the variable ‘breeder’. According to the recommendation of Imdadullah et al. (2016) [22], the three regressors were then excluded from further analysis and only the variables ‘breeder’, ‘sex’ and ‘age at the end of fattening’ remained. Afterwards, a stepwise backward selection of the regressors based on the Akaike information criterion (AIC) value was conducted using olsrr (v0.5.3, [23]). Finally, the relationships between dependent and independent variables were visualized as boxplots using ggplot2 (v3.3.6, [24]).

## Results

### Growth performance, carcass quality and meat quality

As shown in Table 2, growth performance varied between the two batches. Pigs in batch 1 were stalled at a higher age, resulting in shorter fattening periods and lower weight gains. Batch 2 pigs showed larger (daily) weight gains and were heavier at the times of slaughter, although the age of slaughter was comparable between batches.

**Table 2:** Growth performance of two batches of female and castrated Anger Saddleback pigs (n = 58); parameters are expressed as mean ± sd and the coefficient of variance in % (cv).

Batch	1		2	
Number of pigs (n)	40		18	
	mean ± sd	cv	mean ± sd	cv
Age at start of fattening (days)	105.5 ± 21.6	20.5	86.6 ± 23.7	27.4
Age at slaughter (days)	324.9 ± 21.7	6.7	321.6 ± 14.7	4.6
Length of fattening period (days)	219.4 ± 14.3	6.5	235.0 ± 20.4	8.7
Initial live weight (kg)	27.5 ± 7.9	28.8	19.2 ± 8.6	44.6
Final live weight (kg)	139.4 ± 4.7	3.4	152.3 ± 6.0	3.9
Weight gain (kg)	111.8 ± 8.6	7.7	133.1 ± 10.0	7.5
Daily weight gain (g/day)	510.1 ± 28.6	5.6	567.4 ± 28.9	5.1
Carcass weight (kg)	108.9 ± 4.8	4.4	127.6 ± 6.1	4.8
Concentrate intake (as fed) (kg/day)	-	-	1.6 ± 0.5	31.3
Silage intake (as fed) (kg/day)	-	-	2.0 ± 0.1	5.0
Feed conversion (concentrate/gain) (kg/kg)	-	-	3.3 ± 3.3	100

The resulting carcass quality traits are presented in Table 3. Dressing percentage was higher in batch 2 whereas LMP was slightly superior in batch 1. Absolute weights of valuable parts were generally higher in batch 2, which accords with the increased carcass weight, whereas the percentage (weight of the part in relation to carcass weight) was rather similar for both batches. Backfat thickness, in contrast, was much higher in batch 2 as compared to batch 1.

**Table 3:** Carcass quality of two batches of female and castrated Anger Saddleback pigs (n = 58), parameters are expressed as mean ± sd and the coefficient of variance in % (cv).

Batch	1		2	
	mean ± sd	cv	mean ± sd	cv
Carcass weight (kg)	108.9 ± 4.8	4.4	127.6 ± 6.1	4.8
Dressing percentage (%)	78.0 ± 1.8	2.3	83.8 ± 1.8	2.2
Lean meat percentage (%)	47.7 ± 4.6	9.7	45.6 ± 4.8	10.4
Neck (kg)	4.9 ± 0.3	6.1	5.7 ± 0.3	6.1
Neck (%)	4.5 ± 0.3	5.5	4.4 ± 0.2	5.4
Shoulder and thick rib (kg)	10.9 ± 0.6	5.6	12.4 ± 0.9	6.9
Shoulder and thick rib (%)	10.0 ± 0.4	4.1	9.7 ± 0.6	6.5
Loin (kg)	7.9 ± 0.7	8.3	9.2 ± 0.8	8.4
Loin (%)	7.3 ± 0.4	5.7	7.2 ± 0.4	5.6
Tenderloin (kg)	0.7 ± 0.1	13.2	0.8 ± 0.1	12.4
Tenderloin (%)	0.6 ± 0.1	12.3	0.6 ± 0.1	12.5
Belly (kg)	8.4 ± 0.6	6.8	9.9 ± 0.8	7.7
Belly (%)	7.7 ± 0.4	4,9	7.7 ± 0.5	7.0
Ham (kg)	14.4 ± 0.7	5.0	16.2 ± 0.7	4.6
Ham (%)	13.3 ± 0.6	4.4	12.7 ± 0.6	4.8
Backfat thickness (mm)	36.1 ± 5.2	14.4	41.9 ± 6.2	14.8

In terms of meat quality, the pH measured 45 minutes after slaughter in loin and ham (see Table 4) was in a normal range and did not take values below 6.0 indicating a PSE condition of the meat [25]. The limit for pH_24_ of ≤ 6.2 indicating DFD condition [26] was undercut with all results and therefore, a normal acidification is reflected. Electrical conductivity of Loin and ham 24 hours after slaughter was also in a normal range (≤ 7.8 mS/cm, [26]). The Opto-Star values for brightness were excellent for all measurements except for the results after 45 minutes in batch 2, which was still good (< 63 and ≥ 53) [27]. The result for the drip loss in batch 1 does not indicate quality defects, whereas the average drip loss in batch 2 slightly exceeds the limit of 5% [28]. As shown in Table 4, the IMF content was slightly higher in batch 2 with an average result of 2.9% in contrast to 2.5% in batch 1.

**Table 4:** Meat quality characteristics of two batches of female and castrated Anger Saddleback pigs (n = 58); parameters are expressed as mean ± sd and the coefficient of variance in % (cv).

Batch	1		2	
	mean ± sd	cv	mean ± sd	cv
Loin pH 45 minutes	6.2 ± 0.3	4.3	6.2 ± 0.4	5.7
Ham pH 45 minutes	6.4 ± 0.4	5.7	6.1 ± 0.4	6.9
Loin pH 24 hours	5.4 ± 0.1	2.1	5.4 ± 0.2	3.8
Ham pH 24 hours	5.5 ± 0.1	2.5	5.4 ± 0.2	4.5
Loin conductivity 24 hours (mS/cm)	5.2 ± 1.5	29.1	6.8 ± 1.9	28.1
Ham conductivity 24 hours (mS/cm)	4.3 ± 1.6	36.0	7.8 ± 2.6	33.1
Opto-Star value 45 minutes	69.3 ± 7.1	10.3	62.4 ± 12.9	20.7
Opto-Star value 24 hours	71.1 ± 5.6	7.8	68.6 ± 12.2	17.8
Drip loss (%)	4.3 ± 2.3	54.9	6.5 ± 3.9	59.4
Intramuscular fat (%)	2.5 ± 0.7	27.2	2.9 ± 1.0	33.8

### Relationships between variables and effects of influencing variables

**Fig 1:**
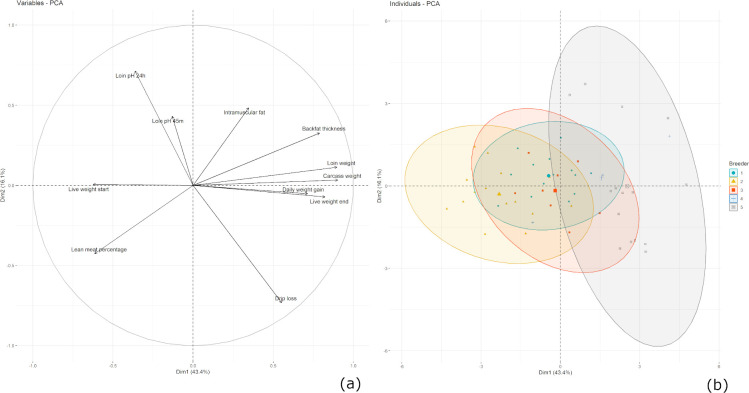
Visualization of principal component analysis of target variables (a) and individuals (n = 58) grouped by breeder (b).

As shown in Fig 1A, the results of the PCA give a first insight in the relationship between the target variables. On the one hand, the first principal component (PC1) explains 43% of the variance, which is due to carcass traits such as live and carcass weight as well as daily weight gain. On the other hand, PC2 is dominated by meat characteristics (e.g. loin pH 45 minutes and 24 hours *post mortem*) and explains 16.1% of the variance. Additionally, it can be drawn from the figure, that IMF and backfat thickness are clearly negatively correlated to LMP. Furthermore, an increase in life weight at the end of fattening is positively correlated with the increase of carcass and loin weight as well as daily weight gain. Additionally, it is shown that the pH value 45 minutes and 24 hours after slaughter is negatively correlated to drip loss (Fig 1A). The second part of Fig 1 shows the PCA of individuals grouped by breeder. From this visualization some signs of clustering regarding the assignment of the animals to their breeder are visible, especially for the animals of breeder 2 and 5 (Fig 1B).

For most of the selected target variables related to growth performance (final live weight, carcass weight, daily weight gain and weight of loin), carcass quality (backfat thickness, LMP) and meat quality (loin pH 45 minutes, loin pH 24 hours and drip loss) significant differences between breeders were found. Only for the IMF and the loin pH value (45 minutes and 24 hours *post mortem*) this influence could not be confirmed in any cases as shown in Table 5. A significant effect of sex was detected for target variables related to carcass quality and meat quality. The age of the pigs at the end of the fattening had a significant influence on the majority of the studied variables except the IMF and loin pH_45_ and pH_24_ (Table 5).

**Table 5:** Associations between independent variables (breeder, sex and age at the end of fattening) and selected target variables related to growth performance, carcass quality and meat quality in Angler Saddleback pigs (n = 58) as assessed in linear regression analyses; parameters are expressed as estimates and related p-values.

Dependent variable		Final live weight (kg)	Carcass weight (kg)	DWG (g/day)	Loin weight (kg)	LMP (%)	BFT (mm)	Loin pH_45_	Loin pH_24_	Drip loss (%)	IMF (%)
Intercept		111.31	70.72	696.89	3.69	69.98	5.05	6.3	5.09	11.5	2.77
Breeder	1	-	-	-	-	-	-			-	-
	2	2.19 (0.24)	-2.05 (0.27)	-16.18 (0.09)	-0.73 (<0.01)	6.17 (<0.01)	-8.63 (<0.01)			-0.65 (0.54)	-0.12 (0.66)
	3	0.60 (0.79)	-0.76 (0.74)	26.27 (0.03)	-0.55 (0.06)	-0.11 (0.94)	-2.73 (0.17)			2.52 (0.07)	0.18 (0.57)
	4	11.83 (<0.01)	16.06 (<0.01)	46.62 (<0.01)	0.76 (0.07)	-0.53 (0.82)	0.71 (0.80)			1.02 (0.58)	-0.25 (0.59)
	5	14.61 (<0.01)	18.48 (<0.01)	57.79 (<0.01)	1.01 (<0.01)	-0.02 (0.99)	2.56 (0.01)			2.78 (0.01)	0.50 (0.07)
Sex	male					-	-	-	-	-	-
	female					1.84 (0.06)		-0.23 (<0.01)	-0.05 (0.22)	1.05 (0.18)	-0.52 (0.01)
Age	(days)	0.08 (0.03)	0.12 (<0.01)	-0.58 (<0.01)	0.01 (<0.01)	-0.08 (0.01)	0.11 (<0.01)		0.00 (0.30)	-0.03	
										(0.25)	
Adj. R-squared		0.6	0.78	0.62	0.54	0.46	0.51	0.14	0.01	0.17	0.14

Estimates (p-values) of parameters; DWG–Daily weight gain, IMF–Intramuscular fat, LMP–Lean meat percentage, BFT–Backfat

**Fig 2:**
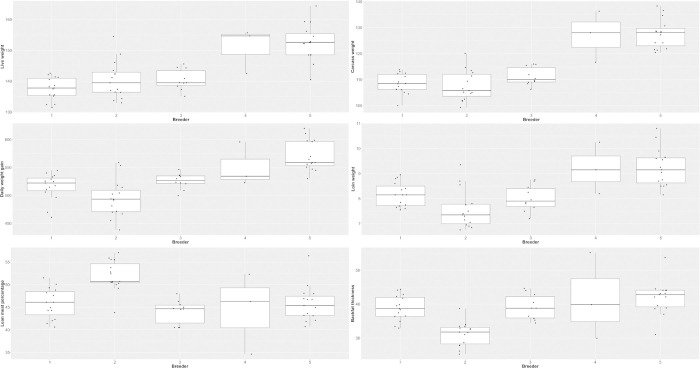
Live weight before slaughter, carcass weight, daily weight gain, loin weight, lean meat percentage and backfat thickness of two batches of female and castrated Angler Saddleback pigs (n = 58) grouped by breeder. Breeders 1 to 3 provided animals for batch 1, breeders 4 and 5 provided animals for batch 2. Data points resemble individual animals, i.e. breeder 4 only provided three pigs.

**Fig 3:**
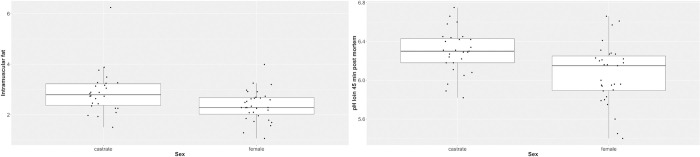
Intramuscular fat and pH_45_ of two batches of Angler Saddleback pigs (n = 58) grouped by sex.

In order to have a more detailed insight in the effects of the influencing variables, a selection of target variables was plotted against the most significant regressor (Figs 2 and 3). As shown in Fig 2, the results of target variables of growth performance as well as LMP and backfat thickness were plotted with distinction of the five breeders. IMF and pH value 45 minutes of loin were presented in Fig 3 with the distinction of results for female and barrows.

## Discussion

In the present study, 58 Angler Saddleback pigs were reared and fattened in single compartments under semi-controlled conditions. Furthermore, a two-phase feeding strategy with slightly reduced energy and protein contents and varying feed amounts was conducted to meet the expected lower nutritional demands of the breed and avoid excessive fat buildup. The number of animals per breeder and per batch as well as the age at the start of the fattening varied greatly. This was due to the overall very small population size and the therefore limited availability of piglets of the same age at the required time. Despite these limitations, the two trials were conducted by the same person in the same manner.

Due to the different age at the start of the fattening, the length of the housing periods varied between 219 days (batch 1) and 235 days (batch 2). By this, a similar age with on average 324 days at the end of the two batches could be realized. However, age at the end of fattening and fattening length was, in comparison to the common practice with modern breeds of around 162 days (age) and 90 days (fattening) [29], more than twice as high. For the majority of results of the three domains growth performance, carcass quality and meat quality, considerable differences between the two batches were found. As a possible explanation, it is important to state that the fattening period of batch 2 was longer and that the age at the start of the project was lower than for the animals in batch 1. This means that the pigs in batch 2 were fed for a longer period, and also in crucial periods of their development, with the grower diet than in batch 1. Furthermore, the husbandry and feeding conditions at the breeder farms, where the rearing took place, might have varied to a large extend. The Angler Saddleback pigs are mainly fed with varying components with less control of nutrient composition [7]. The pigs in batch 1 were therefore exposed for longer to the conditions on their farms of origin, which could therefore have affected their later development. This might be one reason why live and carcass weight at the end of fattening were negatively correlated with the live weight at the start of the fattening period as shown in the visualization of PCA results. The pigs with a lower weight at the beginning of the trials were for a longer time exposed to the conditions (e.g. the feeding regime) in the project and reached therefore higher performance results at the end of fattening.

Regarding growth performance, the Angler Saddleback pigs reached a final live weight of 139 kg (batch 1) and 152 kg (batch 2) with an average daily weight gain of 510 to 567 g per day in the two batches. Angler Saddleback pigs investigated by Brandt et al. (2010) reached a final weight of 116 kg with a distinctly higher daily weight gain of 779 g per day after 117 days of fattening [30], which might be explained by the lower final weight and a different diet composition in that trial. The closely related German Saddleback breed investigated by Nürnberg et al. (2013) reached an average carcass weight of 142 kg with an age of 343 days [31], which was higher than the carcass weight of 109 kg (batch 1) to 128 kg (batch 2) reached herein. The feed conversion herein (3.3 kg concentrate per kg of weight gain on average) was higher than reported for German commercial breeds (2.3 kg, [32]) but comparable with results found by Brandt et al. (2010) for Angler Saddleback pigs [30]. In our calculation, however, nutrient uptake through roughage was neglected, which means that, in this study, true feed conversion was even lower. Above that, the performance traits of the Angler Saddleback pig are partly comparable to other European local pig breeds. For Mangalitsa and Moravka breed from Serbia final live weights between 119 and 131 kg as well as a daily weight gain of 480 g and 545 g are reported [33]. Final live weights of Krškopolje pigs from Slovenia are distinct lower with on average 118 kg at 293 days of age [34].

In view of carcass quality, the assessed LMP was almost similar in the two batches and averaged at 47%. This value is higher than the one reported for German Saddleback pigs (39%, [31]) and Bunte Bentheimer, a comparable local breed also from Germany which reached 43% [35]. Angler Saddleback pig investigated by Brandt et al. (2010) reached an LMP of 49% [30] which is similar to the one found in this study, yet distinctly lower than LMP values of German commercial breeds (61%, [32]). However, the legally recognized methods for the estimation of LMP are intended for maximum carcass weights of 120 kg [17] which was slightly exceeded in batch 2. Furthermore, local pig breeds have certainly played a minor role in the development of the underlying formula of the “two point” method. Therefore, the accuracy of the estimation may have limitations. Backfat thickness in commercial pig breeds has decreased over the last decades due to the focus on LMP in breeding programs, and reaches today 17 mm in commercial German breeds [32]. The mean backfat thickness found in this study was 36 mm (batch 1) and 42 mm (batch 2), and was therefore more than twice as high as in commercial slaughter pigs. This corresponds to the range assessed for Angler Saddleback pigs (36 mm) by Brandt et al. (2010) [30] and for German Saddleback pigs (42 mm) [31]. As for a comparison to other European local pig breeds, with 44% a slightly lower amount of LMP is documented for Krškopolje pigs and in connection with this a slightly higher backfat thickness (38 mm) [34]. The same result for backfat thickness was also reported für Alentejano pig breed from Portugal. In contrast to that for Bisaro, another local pig breed from Portugal, a distinctly lower backfat thickness with 21 mm was found [36]. However, the two Portuguese breeds were slaughtered with a distinctly lower final live weight of 64 kg after 109 days on trial [36].

The meat quality assessments mostly showed no defects in terms of PSE (pale, soft and exudative) meat. The only exception was the drip loss in batch 2, that was with 6.5% slightly higher than the limit of 5% [28]. Additionally, loin and ham conductivity in batch 2 was with 6.8 and 7.8 just slightly below or equal to the cited limit of ≤ 7.8 mS/cm [26]. This indicates a decreased meat quality in comparison to batch 1, although no definite evidence of PSE meat. A reason for that might be inadequate cooling *post mortem* due to delays in the slaughter process. In comparable meat quality analyses of Bunte Bentheimer [35] and German Saddleback pigs [31], no signs of quality defects were found.

A high LMP content is usually associated with lower IMF amounts [37]. Therefore, commercial pig breeds usually reach IMF values below 2% [38]. The Angler Saddleback pigs reached an IMF percentage of 2.6%, which show slight differences between the two batches and also varied between individual animals. Similar IMF levels were found for Duroc pigs [39] and Schwäbisch-Hällisch pigs [40], whereas a distinctly lower IMF of 1.6% was detected for Bunte Bentheimer pigs [35]. For other local pig breeds higher IMF values were reported as for example 3.5% for Krškopolje pigs [34] and 5.1% for heavy Iberian pigs [41]. Bisaro and Alentejano breed reached an IMF of 5.5% and 6.7% [36]. In summary, the results provide further evidence that Angler Saddleback pigs show moderate increased IMF amounts compared to commercial pig breeds, with simultaneously a distinct higher backfat thickness and a lower LMP. In particular, the high IMF may be responsible for its praised sensory characteristics. However, further research is needed to substantiate these claims on improved palatability of Angler Saddleback pigs.

The analysis of influencing variables revealed an effect of breeder reflecting the origin of the piglets on the majority of target variables. The visualization of the target traits, with the distinction between the breeders, especially for live and carcass weight, showed differences between batch 1 and 2. In batch 2, where pigs came from breeder 4 and 5, the animals reached significantly higher live and carcass weights than the pigs in batch 1 (breeders 1 to 3). This was also the case for daily weight gain and loin weight, albeit with a less strong effect. Possible reasons for the differences between the batches have already been described in connection to the longer fattening period and the lower age at the start of the trial in batch 2. The visualization of LMP shows the highest results for pigs from breeder 2, although these animals had on average the lowest means for performance traits (e.g. daily weight gain and loin weight). As it is to expect the backfat thickness was lowest in the latter group (breeder 2) while being higher in the other groups from the remaining breeders. Differences between the animals from different breeders within one batch may reflect genetic heritage as well as husbandry conditions and management before the start of the fattening trial. The former may be related to the breeding management of Angler Saddleback pigs, which is carried out by small groups of breeders and may not be sufficiently coordinated in terms of breeding objectives, selection criteria, exchange of genetic resources etc. This could be also an explanation for the differences even among the pigs from one breeder. In summary, the present results indicate large phenotypic differences in economically important production traits (cf. the high standard deviations and partly high coefficients of variation in Tables 2–4). A similar variation is also reported for German Saddleback pigs [31].

A significant effect of sex on IMF was assessed, whereby barrows showed slightly higher IMF contents. The same effect of sex on IMF content of various pig breeds has been reported before [42–44]. The average pH_45_ of female pigs was slightly lower and thus in a less favourable range with regard to meat quality. This effect has not been detected in other studies [45, 46]. In summary, the detected effects of sex on the different parameters are partly supported by previous studies especially in view of the IMF. On the other hand, comparable studies have uncovered correlations that could not be confirmed from the present data. Among these is the reported effect of sex on backfat thickness and on final live weight [47] as well as on LMP [48].

All selected performance traits as well as carcass quality variables (except IMF) were significantly influenced by the age at slaughter. Pigs that were slaughtered at a higher age, had a higher live and carcass weight, as was expected. This also holds for loin weight and backfat thickness. LMP, on the other hand, decreased due to increasing fatness which is due to allometric growth of lean and fat tissue. These correlations are well documented and can be found, among others, in a review by Wood et al. 2008 [37].

## Conclusions

The assessment of performance, carcass quality and meat quality of 58 purebred Angler Saddleback pigs gives a current-day insight in the phenotypic characteristics of this endangered local breed. As a main result, the data confirmed the Angler Saddleback’s status as a slower growing and thus fatty breed especially in view of its growth performance, LMP and backfat thickness. The overall higher amount of fat in the carcass and meat of the Angler Saddleback pigs may be responsible for the praised sensory characteristics of the meat. Although the IMF cannot be classified as particularly high, especially with regard to other local European pig breeds. The detected significant influence of the independent variable “breeder” on most of the target variables revealed a variability of the target variables between the pigs. Beyond the certain variation between the two batches, this indicates differences in husbandry and feeding conditions at the rearing farms, as well as a possible genetic variation within the breed. The latter may be caused by the less consideration of breeding objectives and selection criteria in the management of the breed. Further investigations are necessary to better distinguish between these influencing variables.

With regard to the practical importance, the presented results shed light on the properties of the Angler Saddleback pig which were previously largely unproven attributions. Among others this forms the base to develop suitable meat products with fitting marketing concepts especially in view of the reduced LMP. This in turn is an important step to make the breed profitable which is a sustainable strategy for the conservation of local breeds [49].
